# Making the most of peer review

**DOI:** 10.7554/eLife.12708

**Published:** 2015-11-11

**Authors:** Nikolai Slavov

**Affiliations:** Department of Bioengineering, Northeastern University, Boston, United Statesnslavov@alum.mit.edu

**Keywords:** scientific publishing, peer review, post-publication peer review, open science, reproducibility

## Abstract

Journals should publish referee reports and respond to well-founded concerns about papers after publication.

The scientific community spends a significant amount of time on the peer review of manuscripts submitted to journals. Many of the referee reports written during this process contain insights, comments, suggestions and questions that help authors to improve their manuscripts. However, with the exception of a small number of journals, these reports are only ever seen by the authors of the manuscript and the editors of the journal. I read referee reports when they are available, and I find them to be very helpful. Hiding them is an enormous waste.

Before journals moved onto the Web in the 1990s, anyone who wanted to comment on a published paper had to publish another paper in response, a process taking many months or even years. Some journals had special sections for short articles that challenged papers published in the journal, but many of these journals seemed reluctant to publish such challenges (and they took a long time to publish those challenges that were accepted for publication). Over the past decade, however, researchers have turned to other platforms to comment on and criticize papers that have been published in journals. These platforms include blogs and third-party websites such as PubMed Commons and PubPeer. A number of journals also allow readers to post comments at the end of articles.

As with referee reports, I often read blogs and comments and find many—but not all—of them helpful. However, just as the scientific community is failing to take full advantage of the time and expertise that goes into referee reports, I feel that we are also failing to take advantage of the possibilities offered by post-publication comments on papers; indeed, many legitimate comments remain ignored by authors, journals and universities (PubPeer, 2014). By neglecting these comments we are missing an opportunity to reduce the time and resources that are spent trying to repeat and build on experiments that are not reproducible (Freedman et al., 2015).

I often read blogs and comments and find many—but not all—of them helpful.

What is the evidence that post-publication comments are helpful? Three recent scientific controversies—the report that a bacterial DNA sample contains arsenic instead of phosphorous, the report that RNA sequences contain widespread edits/changes from the corresponding DNA sequences, and the report of stimulus-triggered acquisition of pluripotency (STAP)—show that post-publication comments offer a fast and efficient method for raising concerns about potential problems in published papers. Criticisms of the arsenic life paper were first raised on blogs shortly after it was published online in *Science* in December 2010 (Wolfe-Simon et al., 2011), and the journal subsequently published eight Technical Comments expressing concern about the paper in May 2011. Bloggers started to ask questions about the RNA paper, which was published in *Science* in May 2011 (Li et al., 2011), shortly after publication (Pickrell, 2011), and the journal subsequently published three Technical Comments about the paper in March 2012 (Hayden, 2012). Concerns about the two STAP papers, which were published in *Nature* in January 2014 (Obokata et al., 2014a, 2014b), were raised immediately on PubPeer and blogs (The Niche, 2014), and both papers were retracted in July 2014.

These three examples show the potential of post-publication comments as a force for good (and also raise the worrying question about the way that papers with such obvious flaws were able to withstand peer review). There have also been lengthy and sometimes heated/acrimonious debates on blogs about papers on topics as diverse as network inference/deconvolution algorithms (Kellis, 2014; Pachter, 2014) and stripy nanoparticles (Stirling, 2015). Since these debates are not moderated and do not result in any sort of conclusion, they can be confusing for readers who are not experts in the subject being discussed. As a result, as someone who has developed network inference algorithms, I am often asked for my opinion on post-publication debates in this area. I believe that input from editors would be of great value in such cases.

Of course, every post-publication comment on a paper does not have to lead to the publication of a formal challenge (such as a Technical Comment in *Science* or a Brief Communication Arising in *Nature*) or a retraction. However, I feel that many of the legitimate concerns about papers that are being raised in blogs and other platforms are being ignored by journals, so there is a clear need to make sure that comments that satisfy some basic criteria (see below) are acted upon by journals. The peer review process, the reproducibility of published results, and the scientific community as a whole stand to benefit tremendously from a more inclusive consideration of post-publication comments. Here I propose how this might work.

Legitimate concerns about papers raised on blogs and other platforms are being ignored by journals.

First, journals should publish referee reports, and referees should be encouraged (but not required) to sign their reports. This would provide useful context about the paper (such as scientific caveats that are not reflected in the binary accept or reject decision) and would also, I am sure, lead to better reports. It would also give readers more insight into the quality and rigor of the peer review processes at different journals.

Second, journals should agree to consider non-anonymous post-publication comments submitted to certain platforms within a certain timeframe after the paper has been published. This timeframe could be as short as a few weeks or a month. Journals would be obliged to publish a response from the authors to all the substantive concerns contained in the comments. Concerns that would require a response would include the following:

-crucial controls that are missing
-major inconsistencies between the data and the main conclusions
-inconsistencies with the published literature that are not discussed in the paper
-failures by readers to reproduce analyses described in the paper
-errors in mathematical proofs.

Comments that would not require a response would include: requests for more data and/or experiments that would extend the scope of the work; concerns over novelty or significance; and questions about issues that were raised, discussed and settled during the peer-review process. (Publishing referee reports would, I am sure, make this process more manageable.)

In the future I believe that journals should also consider anonymous post-publication comments if the commenter is prepared to reveal their identity to the journal editor in confidence. However, this is likely to be controversial so, in the first instance, I think we should focus on getting journals to consider non-anonymous comments. I would also encourage researchers who comment on papers on blogs and other platforms (such as PubPeer) to post their comments (or links to them) on PubMed Commons and on the paper itself (if comments are allowed).

Publishing referee reports and requiring journals to respond to substantive post-publication comments would, I believe, bring benefits to the scientific literature and to researchers who make constructive comments. Authors and journals alike might be reluctant to commit to spending more time on a paper that has been published, especially if the paper had to be revised a number of times during the peer review process, but both have to take responsibility for the research they publish, and this should extend to dealing with substantive and legitimate questions and concerns. Online post-publication discussions about papers are becoming more frequent, and I believe this trend will continue. Authors and journals must get involved in these discussions for the benefit of all concerned.
